# Identification of tubular injury microRNA biomarkers in urine: comparison of next-generation sequencing and qPCR-based profiling platforms

**DOI:** 10.1186/1471-2164-15-485

**Published:** 2014-06-18

**Authors:** Rounak Nassirpour, Sachin Mathur, Mark M Gosink, Yizheng Li, Ahmed M Shoieb, Joanna Wood, Shawn P O’Neil, Bruce L Homer, Laurence O Whiteley

**Affiliations:** Drug Safety, Pfizer Worldwide Research and Development, 1 Burtt Rd, Andover, MA 01810 USA; Business Technology, Pfizer Research and Development, Burtt Rd, Andover, MA 01810 USA; Drug Safety, Pfizer Research and Development, MS 8274 E. Point Road, Groton, CT 06340 USA; Quintiles Drug Research Unit at Guy’s Hospital, 6 Newcomen St, London, SE1 1YR UK; 200 Cambridge Park, Cambridge, MA 01810 USA

**Keywords:** Next-generation sequencing, NGS, Small RNA, TruSeq, qRT-PCR, Urine, miRNA, Gentamicin, Kidney injury

## Abstract

**Background:**

MicroRNAs (miRNAs) are small, non-coding RNAs that regulate protein levels post-transcriptionally. miRNAs play important regulatory roles in many cellular processes and have been implicated in several diseases. Recent studies have reported significant levels of miRNAs in a variety of body fluids, raising the possibility that miRNAs could serve as useful biomarkers. Next-generation sequencing (NGS) is increasingly employed in biomedical investigations. Although concordance between this platform and qRT-PCR based assays has been reported in high quality specimens, information is lacking on comparisons in biofluids especially urine. Here we describe the changes in miRNA expression patterns in a rodent model of renal tubular injury (gentamicin). Our aim is to compare RNA sequencing and qPCR based miRNA profiling in urine specimen from control and rats with confirmed tubular injury.

**Results:**

Our preliminary examination of the concordance between miRNA-seq and qRT-PCR in urine specimen suggests minimal agreement between platforms probably due to the differences in sensitivity. Our results suggest that although miRNA-seq has superior specificity, it may not detect low abundant miRNAs in urine samples. Specifically, miRNA-seq did not detect some sequences which were identified by qRT-PCR. On the other hand, the qRT-PCR analysis was not able to detect the miRNA isoforms, which made up the majority of miRNA changes detected by NGS.

**Conclusions:**

To our knowledge, this is the first time that miRNA profiling platforms including NGS have been compared in urine specimen. miRNAs identified by both platforms, let-7d, miR-203, and miR-320, may potentially serve as promising novel urinary biomarkers for drug induced renal tubular epithelial injury.

## Background

There currently are no biomarkers that can accurately track, correlate, or predict the occurrence of renal injury, particularly when related to drug-related toxicity [1–3]. There is a growing interest in the role of micro Ribonucleic Acids (miRNAs) in the pathogenesis of renal diseases [4, 5] and a growing number of investigations are directed to characterizing their potential as biomarkers [6–9]. miRNAs are highly conserved, endogenous, small (19–25 nucleotides), non-coding RNAs, with the primary role implicated in post-transcriptional silencing. Since a single miRNA can regulate multiple transcripts, miRNAs can potentially impact multiple signaling pathways and their dysregulation may lead to a variety of different disorders including renal disease [10]. In fact, several miRNAs have been implicated in various renal diseases, including diabetic nephropathy, hypertension, glomerulonephritis, renal cancer, and polycystic kidney disease [11–17]. miRNAs are secreted into the circulation by many different mechanisms including various lipid-containing vesicles, such as exosomes, microvesicles, and apoptotic bodies, but can also be protected from degradation by binding to RNA-binding proteins [9]. Due to their stability, miRNAs are readily quantified in serum, plasma and other body fluids, and thus more investigations are being directed to characterizing their potential as biomarkers. In fact, study results from our laboratory and other participating members of the Health and Environmental Sciences Institute’s (HESI) Nephrotoxicity Committee have identified changes in miRNA levels in various kidney disease animal models. Although quantitative real-time PCR (qRT-PCR) has most commonly been used to profile annotated miRNome, newer technologies which promise increased sensitivity and specificity are becoming available at astonishing rates. For example, next-generation sequencing (NGS) is gaining popularity and has successfully been used to characterize miRNA profiles in various tissues [18] as well as bio-fluids including blood and, most recently, cerebral spinal fluid [19, 20]. Although NGS platform has been shown to generate highly reproducible, accurate data with high correlation to other platforms for high quality RNA samples [21–24], comparative data on media with low quality and quantity RNA are lacking.

The objective of this study was to characterize the miRNA expression changes using two different profiling platforms in urine specimens following induction of toxic renal tubular injury in rodents with gentamicin. Gentamicin is an aminoglycoside antibiotics which like other nephrotoxicants such as mercuric chloride and chromium induces injury of renal tubular epithelial cells [25]. Gentamicin has also been reported to induce more severe nephrotoxicity than other aminoglycoside antibiotics when administered at high doses [26, 27] and has already been shown to induce miRNA changes in rats, albeit at a different dose and treatment regimen [28]. Accordingly, we performed a global urinary miRNA expression profiling using both the more conventional qRT-PCR TaqMan Low Density Array (TLDA-A, Life Technologies) method as well as NGS in control and treated rats. The presence of tubular epithelial injury was confirmed by histopathology and assessment of a panel of urine protein biomarkers that have been qualified for use in non-clinical drug development [29]: kidney injury molecule-1 (KIM-1), total protein, and beta 2- microglobulin.

Although a recent paper has described NGS characterization of exosomal and non-exosomal miRNAs in human urine [30], to our knowledge, this is the first time that the NGS and qRT-PCR based platforms have been compared in urine. We report that although both platforms were able to identify drug induced changes in miRNA expression, that there is discordance in the level of sensitivity of detection. Specifically, with exception of three miRNAs, miRNA-seq did not detect some miRNAs which were identified by qRT-PCR with threshold cycle (Ct) values lower than 32 in every urine specimen analyzed. These differences could be due to the level of sensitivity posed by NGS analysis for low abundant miRNAs which, are easily detected by the qRT-PCR method (performed with pre-amplification). On the other hand, the vast majority of miRNAs detected by NGS were in their isomeric forms, and thus could not have been detected by the pre-designed qRT-PCR primers available on the TLDA-A. Our results suggest that although miRNA-seq is specific, with few false positive calls, it may not detect certain low abundant miRNAs in low quality RNA specimen like those currently obtained from urine. However, the qRT-PCR analysis will miss a wealth of information, most importantly in the form of isoforms. In summary, we have compared the detection of urinary miRNA alterations in tubular injury with qRT-PCR and miRNA-seq. Each platform has its strengths and weaknesses and therefore, careful considerations should be made when selecting a platform of choice for urinary miRNA profiling.

## Results

### Histopathology and urinary protein biomarker data indicate gentamicin induced tubular injury at D7 with 50 mg/kg

**Figure 1:**
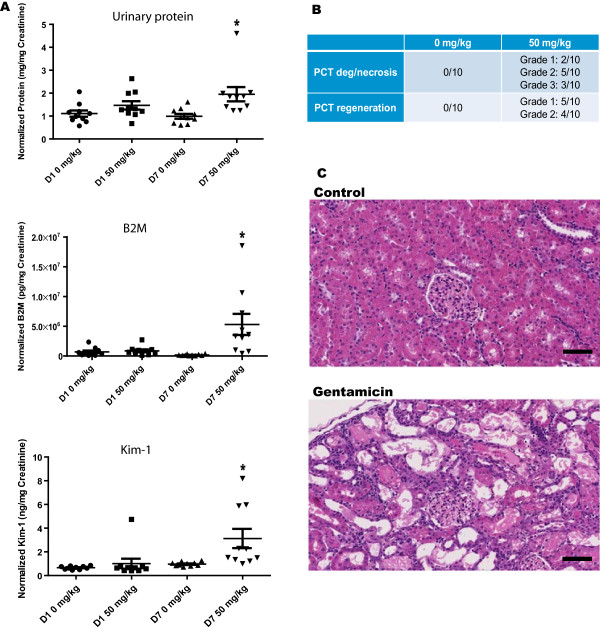
**Gentamicin-induced nephrotoxicity in rats. (A)** Urinary protein, β2-microglobulin (B2M), and Kim-1 (all normalized to urine creatinine), indicate tubular injury at D7 (Mean +/- SD; *denotes p value < 0.05). **(B)** Histopathology assessment of the kidneys revealed moderate degeneration and necrosis with evidence of regeneration of the PCT at day 7. **(C)** Section of kidney with moderate degeneration and necrosis of the PCT at day 7; Control (0 mg/kg/day), top and 50 mg/kg/day bottom. 10 X, H&E stain. Scale bar = 200 microns.

In order to characterize the miRNA changes that could be used as biomarkers of acute renal tubular injury, a study was conducted in the context of the HESI committee on Biomarkers of Nephrotoxicity in a well-established rat gentamicin toxicity model. Rats were injected subcutaneously daily for seven days with various dose levels of gentamicin, including 0 and 50 mg/kg/day. Minimal urine biomarker changes were observed after only 24 hours of dosing. However, after 7 days of dosing, there were significantly increased levels of urine total protein, Beta-2-Microglobulin and Kim-1 (Figure 1A), consistent with renal tubular injury.These urine biomarker levels correlated well with the histopathological findings, which involved proximal convoluted tubules (PCT), and to a lesser extent, distal convoluted tubules (DCT). These changes consisted of tubular epithelial degeneration and necrosis characterized by varying degrees of vacuolar and hyaline degeneration, attenuation, loss of epithelial cellular detail (necrosis), and detachment from intact basement membranes. Cellular debris and cellular and hyaline casts were present in tubular lumens (Figure 1B-C). These changes were associated with multifocal minimal to mild tubular epithelial regeneration occurring primarily in the PCT, and were characterized by basophilic cytoplasm, vesicular nuclei, and infrequent mitoses. In three rats, PCT degeneration and necrosis was categorized grade 3 (moderate) at Day 7 (Figure 1B) and hence they were selected for further miRNA analysis in the NGS studies. There were minimal to mild interstitial mononuclear leukocytic infiltrates. These changes correlated with increased organ weight and macroscopically pale/enlarged kidneys.

### Levels of miRNAs in urine significantly changed following gentamicin induced tubule injury

**Figure 2:**
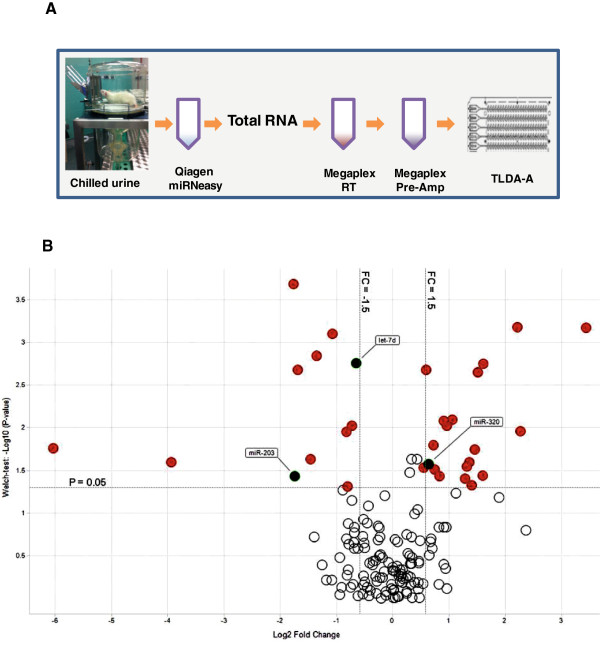
**miRNA expression profiling using Taqman qRT-PCR reveals several significantly changed miRNAs in urine specimens following gentamicin induced renal injury. (A)** Workflow of qRT-PCR analysis. **(B)** Volcano plot shows 32 miRNAs that are significantly regulated (red) at day 7 after gentamicin treatment (Welch test P-value < 0.05 and FC > 1.5 either direction). miRNAs also detected by NGS are labeled. Horizontal line: P-value 0.05; vertical lines: FC at -1.5 and 1.5. Data are normalized using lowess normalization.

The transcript levels of 375 miRNAs were measured in the urine specimen for two time points (day 1 and day 7) after gentamicin treatment by TLDA-A (Figure 2A). 178 miRNAs were detected by qRT-PCR with C_t_ values of ≤ 32 in at least 20% of the urine specimen analyzed. It is important to note that although 178 miRNAs were actually detected in urine by qRT-pCR only 173 (118 in rat, 54 in mouse and 1 in human) had a corresponding identifier in mirBase v 20. Similarly, although 32 urinary miRNAs changed significantly with gentamicin treatment (>1.5-fold and p < 0.05) seven days post treatment as compared to the controls (Figure 2B, Table 1) and all were identified in mirBase v 20. A total of 20 miRNAs were up-regulated and 12 were down-regulated. Concordant with the urinary biomarkers analyzed, no significant miRNA changes were observed at day 1 post gentamicin treatment (data not shown). Furthermore, no significant gentamicin induced changes were observed in urine volumes (control: 18.55 ml ± 7.96; Treated: 21.44 ml ± 5.05).

**Table 1:** **Of the 173 miRNAs identified in urine specimens by qRT-PCR, 32 miRNAs changed significantly due to treatment with p-values < 0.05 and ≥ ±1.5 fold-change**

Detector name (ABI)	Assay ID	miRNA ID v. 20	P-value	FC	miRNA Sequence
mmu-miR-134-4373299	001186	rno-miR-134-5p	0.001	10.83	UGUGACUGGUUGACCAGAGGGG
mmu-miR-342-3p-4395371	002260	rno-miR-342-3p	0.011	4.83	UCUCACACAGAAAUCGCACCCGU
mmu-miR-494-4395476	002365	mmu-miR-494-3p	0.001	4.65	UGAAACAUACACGGGAAACCUC
rno-miR-207-4381096	001315	mmu-miR-207	0.002	3.06	GCUUCUCCUGGCUCUCCUCCCUC
mmu-miR-345-3p-4395659	002529	mmu-miR-345-3p	0.036	3.03	CCUGAACUAGGGGUCUGGAGAC
mmu-miR-193b-4395597	002467	mmu-miR-193b-3p	0.002	2.84	AACUGGCCCACAAAGUCCCGCU
mmu-miR-34a-4395168	000426	rno-miR-34a-5p	0.018	2.74	UGGCAGUGUCUUAGCUGGUUGU
rno-miR-381-4381102	001322	mmu-miR-381-3p	0.047	2.65	UAUACAAGGGCAAGCUCUCUGU
mmu-miR-208-4373091	000511	mmu-miR-208a-3p	0.025	2.57	AUAAGACGAGCAAAAAGCUUGU
mmu-miR-218-4373081	000521	rno-miR-218a-5p	0.028	2.49	UUGUGCUUGAUCUAACCAUGU
mmu-miR-185-4395382	002271	rno-miR-185-5p	0.039	2.44	UGGAGAGAAAGGCAGUUCCUGA
mmu-miR-125b-5p-4373148	000449	rno-miR-125b-5p	0.008	2.08	UCCCUGAGACCCUAACUUGUGA
mmu-miR-140-4373374	001187	rno-miR-140-5p	0.009	1.94	CAGUGGUUUUACCCUAUGGUAG
mmu-miR-16-4373121	000391	rno-miR-16-5p	0.008	1.88	UAGCAGCACGUAAAUAUUGGCG
mmu-miR-489-4378114	001302	mmu-miR-489-3p	0.037	1.78	AAUGACACCACAUAUAUGGCAGC
mmu-miR-208b-4395401	002290	mmu-miR-208b-3p	0.031	1.68	AUAAGACGAACAAAAGGUUUGU
mmu-miR-409-3p-4395443	002332	mmu-miR-409-3p	0.016	1.65	GAAUGUUGCUCGGUGAACCCCU
mmu-miR-320-4395388	002277	rno-miR-320-3p	0.027	1.56	AAAAGCUGGGUUGAGAGGGCGA
mmu-miR-186-4395396	002285	rno-miR-186-5p	0.002	1.5	CAAAGAAUUCUCCUUUUGGGCU
mmu-miR-574-3p-4395460	002349	mmu-miR-574-3p	0.029	1.46	CACGCUCAUGCACACACCCACA
mmu-let-7d-4395394	002283	rno-let-7d-5p	0.002	-1.57	AGAGGUAGUAGGUUGCAUAGUU
mmu-miR-106b-4373155	000442	rno-miR-106b-5p	0.009	-1.65	UAAAGUGCUGACAGUGCAGAU
mmu-miR-363-4378090	001271	mmu-miR-363-3p	0.048	-1.74	AAUUGCACGGUAUCCAUCUGUA
mmu-miR-34b-3p-4395748	002618	rno-miR-34b-3p	0.011	-1.76	AAUCACUAACUCCACUGCCAUC
mmu-miR-23b-4373073	000400	rno-miR-23b-3p	0.001	-2.1	AUCACAUUGCCAGGGAUUACC
mmu-miR-182-4395729	002599	rno-miR-182	0.001	-2.56	UUUGGCAAUGGUAGAACUCACACCG
mmu-miR-30b-4373290	000602	rno-miR-30b-5p	0.023	-2.76	UGUAAACAUCCUACACUCAGCU
mmu-miR-146a-4373132	000468	rno-miR-146a-5p	0.002	-3.21	UGAGAACUGAAUUCCAUGGGUU
mmu-miR-203-4373095	000507	rno-miR-203a-3p	0.037	-3.33	GUGAAAUGUUUAGGACCACUAG
mmu-miR-200c-4395411	002300	mmu-miR-200c-3p	0.000	-3.41	UAAUACUGCCGGGUAAUGAUGGA
mmu-miR-302b-4378071	000531	mmu-miR-302b-3p	0.025	-15.27	UAAGUGCUUCCAUGUUUUAGUAG
mmu-miR-19a-4373099	000395	rno-miR-19a-3p	0.017	-65.54	UGUGCAAAUCUAUGCAAAACUGA

### Small RNA Next Generation Sequencing can be used to evaluate miRNA expression in urinary samples

**Figure 3:**
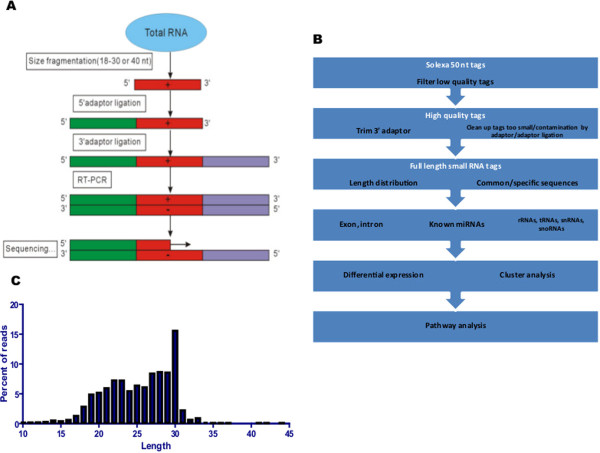
**Workflow for sequencing and analysis of miRNA changes. (A)** Flowchart depicting miRNA sample preparation and sequencing. After extracting the total RNA from the samples, gel select Small RNA (18 ~ 30 nt), 5' RNA adapter ligation and gel purification, 3' RNA adapter ligation and gel purification, and RT-PCR and gel purification, the library products were ready for sequencing analysis via Illumina HiSeqTM 2000. **(B)** Flowchart depicting bioinformatic processing and analysis. After sequencing, raw reads were cleaned by removing low quality reads and short reads. Reads were profiled by mapping them to miRBase v. 20 and other sequence databases. **(C)** Summary (percent) of size distribution of small RNA sequences in the urinary samples analyzed.

**Figure 4:**
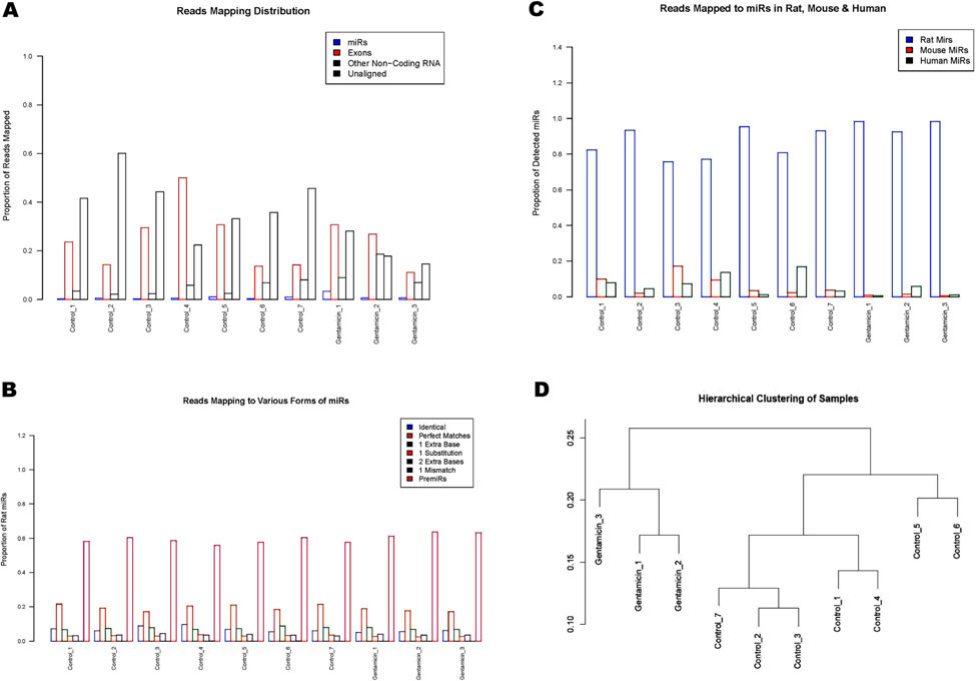
**Characterization of miRNAs using NGS. (A)** Y-axis represents proportion of total reads. Labels:”miRs”-Reads mapping to miRNAs from rat, mouse and human, “Exons”-Reads mapping to rat exons, “Other Non-Coding RNA”-Reads mapping to Non-coding RNA (except miRNAs), “Unaligned”-Reads that could not be aligned to rat genome. **(B)** Y-Axis represents proportion of the total reads that map to all rat miRNAs (mature + isomiRs). “Identical”-Reads that are identical to miRNAs, “Perfect Matches”- Reads that are shorter than miRNAs, “1 Extra Base”-Reads that have an extra base on 3’ and 5’, “1 Substitution”- Reads that have a substitution on 3’ and 5’, “2 Extra Bases”-Reads that have 2 extra bases on 3’ and 5’, “1 Mismatch”-Reads that have at most 1 mismatch (does not include the previous 1 substitution/deletion/insertion), “PremiRs”-Reads that map the precursor miRNA. **(C)** Y-Axis gives the proportion of all miRNAs (rat + human + mouse) with maximum value of 1. The rat, mouse and human miRNAs include mature + isomiRs including precursor miRNAs. **(D)** Hierarchical clustering of the samples. Spearman correlation coefficient was used and the method used was “complete”, using the maximal separation.

We next carried out Next Generation sequencing (NGS) to assess the feasibility of this new technology for analyzing miRNAs in urine samples as compared to the traditional more prevalent qRT-PCR methods widely used. These cross-comparison studies were performed on seven control urine samples collected at day 7 from rats with no tubular injury as well as the three rats from the 50 mg/kg group from day 7 with the highest (grade 3) level of gentamicin induced tubular injury (Figure 1B). As depicted in Figure 3A, after isolating total RNA from the urine specimen and checking for RNA extraction quality as well as quantity, half of each extraction was used in the TLDA analysis and the other half was sent to Beijing Genomics Institute of Americas Corporation (BGI, China) for small RNA sequencing. Table 2 lists the quality and quantity of the RNA extracted from the urine specimens and depicts the poor quality as well as low quantity in the 7 control and 3 gentamicin treated samples. RNA samples were further processed, including gel selection of Small RNAs (18 ~ 30 nt), 5' RNA adapter ligation and gel purification, 3' RNA adapter ligation and gel purification, RT-PCR and gel purification, and despite their low quality did yield a comprehensive small RNA library which was used in the sequencing via the Illumina HiSeqTM 2000 (Figure 3A).The NGS analysis on the control (n = 7) and gentamicin treated (n = 3) urine specimens, yielded an average of 24 million raw reads/sample. After removing adapter sequences and filtering out reads too short to be accurately mapped (less than 18 nucleotides), we obtained on average 22 million clean reads/urine sample (Figure 3B). On average 99% of the sequences were trimmed and ~96% were between 16 and 35 nucleotides long (Figure 3C). Figure 4A shows the breakdown of miRNAs, exons, non-coding RNA (excluding miRNAs) and unaligned sequences. The miRNAs formed a very small portion of reads ranging from 0.1-3% of total reads. As explained in the methods section, the mapping is dominated by precursors (>60%), which indicates a lot of heterogeneity in the miRNAs not accounted for by base substitutions/deletions/insertions at 3’ and 5’ ends. The vast majority of miRNAs detected in these urine specimens were in fact isomiRs (Figure 4B). We also observed that there were negligible amounts of primer-dimers (<3%, data not shown). Exons constituted 11% to as much as 50%. 2-19% of the reads mapped to other non-coding RNAs including snoRNA, rRNA, lincRNA, while a major portion of the reads (14-60%) remained unaligned in the 10 samples and perhaps could be indicative of contaminants. We next investigated the alignment of the reads to other species. Figure 4C illustrates that although a substantial portion of miRNAs aligned to the rat (75-98%), 0.6-17% aligned to the mouse genome while 0.6-16% mapped to human. Specifically, 227 distinct species of miRNAs were detected: 183 from rat, 35 from mouse and 9 from human.We next moved to the cluster and differential expression analysis (Figure 4B). We observed that clustering between the groups was lost when the filtering criteria were altered and we thus had to employ various filtering criteria to optimize the segregation between control and gentamicin treated samples. This lack of a clear segregation suggests that very few miRNAs are differentially expressed between treated and control samples and that the miRNAs are expressed at very low levels. Filtering criterion allowing for inclusion of miRNAs which were detected in at least 2 of 10 samples gave the best segregation (Figure 4D). This yielded 146 miRNAs (121 from rat, 23 from mouse and 2 from human). When we compared the 146 miRNAs detected by NGS to the 173 miRNAs identified by qRT-PCR, we observed that although 60 were detected by both platforms, 87 were detected only by NGS and 111 were specific to TLDA-A.

**Table 2:** **Urine samples from control (samples 1–7) and gentamicin treated rats (samples 32, 33, 39) were used for RNA isolation using the miRNeasy (Qiagen) extraction kit and analyzed for quality and quantity before NGS analysis**

Sample	Gentamicin (mg/kg)	Conc. (ng/ul)	OD 260/280	OD 260/230	RIN
1	0	26.4	1.48	0.26	2.0
2	0	42.7	1.57	0.29	2.6
3	0	41.1	1.53	0.2	2.5
4	0	31.1	1.5	0.1	2.2
5	0	41.5	1.61	0.22	2.6
6	0	28.0	1.51	0.23	1.8
7	0	54.1	1.65	0.32	5.4
32	50	44.9	1.71	0.57	2.6
33	50	46.3	1.6	0.18	2.5
39	50	56.9	1.66	0.7	2.6

Additionally, we also investigated the extent of concordance as the percentage of rat miRNAs that are detected in the control and gentamicin-treated samples analyzed by both NGS and TLDA-A (Table 3). Out of the total 132 rat-miRNAs detected in TLDA-A 48.52% (64) are detected in all 7 control samples. Similarly, out of the 168 NGS detected rat miRNAs 22.02% (37) were detected consistently in all control samples. Thirty five out of the thirty seven miRNAs consistently detected in all 7 controls by NGS were also detected in all 7 samples by TLDA-A. However, 33.33% (55) are detected in only 1 sample in NGS compared to only 7.87% (10) in TLDA-A. Similar investigations from our NGS analysis show that out of the 183 miRNAs detected, 40% (73) occur in only isomeric forms and do not have a mature form in miRBase v20. Twenty one of the seventy three isomeric miRNAs are detected in 7 samples, whereas 49 (67%) are detected in only one sample. Interestingly six of these isomeric miRNAs could potentially also be detected by TLDA-A as the primers used in the platform may not distinguish these from their mature forms. Out of 97 miRNAs with mature forms, 27 are detected in all 7 control urine specimen. Similar trends are observed in gentamicin-treated samples.

**Table 3:** **Percentages of miRNAs detected in control (7) and gentamicin-treated (3) specimen**

Sample		1	2	3	4	5	6	7
Control	NGS	33.33	16.66	5.35	8.33	8.33	5.95	22.02
	TLDA	7.87	6.30	6.30	7.87	7.09	15.75	48.82
Gentamicin	NGS	32.75	19.87	47.41				
	TLDA	19.23	16.15	64.62				

Since a large proportion of reads (14-60%) remained unaligned to rat (Figure 4A), we investigated the origin of highly represented sequences in the reads that remained unaligned. In 2 of the 3 gentamicin treated specimen, a single sequence comprised over 40% of the unaligned reads. One sample was highly contaminated by a PCR primer and the other sample was contaminated with a sequence from the zebrafish. Similarly, in control samples we observed contamination from a variety of bacterial and fungal genomes (e.g. from *Saccharomyces cerevisiae, Paenibacillus polymyxa, Ethanoligenens harbinense, Roseburia hominis,* to name a few). This may indicate fungal and bacterial contamination during sample collection or alternatively these contaminations may have been inadvertently introduced during sample processing procedures. To what extent these contaminations affected the NGS results needs to be determined although among control samples, sample 4 had the least amount of contamination and detected the maximum number (111) of distinct miRNA species, whereas sample 2 that had the most contamination contributed 76 miRNAs.

We also investigated the possibility that some of these unaligned reads may have a dietary origin and thus performed additional mapping to the wheat, corn and soybean genomes. We observed that a proportion of our unaligned reads did originate from dietary genomes from wheat (2%-52%, mean = 19%), corn (0.1%-3.8%, mean = 1.2%) and soybean (0.1%-15%, mean = 3.8%).

Next we investigated various normalization strategies. Recent literature suggest that miRNA-Seq has its own challenges and applying the normalization strategies commonly used in mRNA-seq may need further refinement for use on miRNA sequencing data [31, 32]. We thus compared three different normalization strategies and three differential analysis methods. DESeq and EdgeR were applied to their respective normalizations and Limma was applied to RPM normalized data. EdgeR normalized values gave the best segregation and gave the maximum number of overlap with the differentially regulated miRNAs identified by qRT-PCR and hence are reported here.

Applying the above described clustering and normalization strategies resulted in identification of 14 differentially regulated miRNAs (p-value < 0.05 and ≥ 1.5 fold change in expression). 9 were up regulated and 5 were down regulated (Table 4). Three miRNAs, rno-miR-320-3p, rno-miR-203a-3p, and rno-let-7d-5p, were found to be significantly altered by both the qRT-PCR and NGS analysis in the gentamicin treated urine specimens. For these miRNAs, both platforms reported them to be similarly regulated; miR-320 was up regulated while let-7d and miR-203 were down regulated (Tables 1 and 4).

**Table 4:** **Of the 146 miRNAs identified in urine specimens using NGS analysis, 14 had p-values < 0.05 and ≥ ±1.5 fold-change**

miRNA ID	FC	p-value	Sequence
rno-miR-378a-3p	3.1775	0.0002	ACUGGACUUGGAGUCAGAAGG
mmu-miR-5100	-23.7	0.001	UCGAAUCCCAGCGGUGCCUCU
rno-miR-30e-3p	3.9961	0.0031	CUUUCAGUCGGAUGUUUACAGC
rno-miR-125b-2-3p	74.135	0.0049	ACAAGUCAGGCUCUUGGGACCU
rno-let-7d-5p	-177.8	0.0084	AGAGGUAGUAGGUUGCAUAGUU
rno-miR-320-5p	92.957	0.01	GCCUUCUCUUCCCGGUUCUUCC
rno-miR-100-5p	10.010	0.012	AACCCGUAGAUCCGAACUUGUG
rno-miR-203a-3p	-5.428	0.0158	GUGAAAUGUUUAGGACCACUAG
rno-miR-21-5p	2.4576	0.0241	UAGCUUAUCAGACUGAUGUUGA
rno-miR-3473	-3.990	0.0308	UCUAGGGCUGGAGAGAUGGCUA
rno-miR-21-3p	14.377	0.0329	CAACAGCAGUCGAUGGGCUGUC
hsa-miR-7641	-3.095	0.0359	UUGAUCUCGGAAGCUAAGC
rno-miR-320-3p	2.7415	0.0394	AAAAGCUGGGUUGAGAGGGCGA
rno-miR-455-5p	44.539	0.0405	UAUGUGCCUUUGGACUACAUCG

### Prediction of miRNA targets and pathway analysis relevant to renal injury

**Figure 5:**
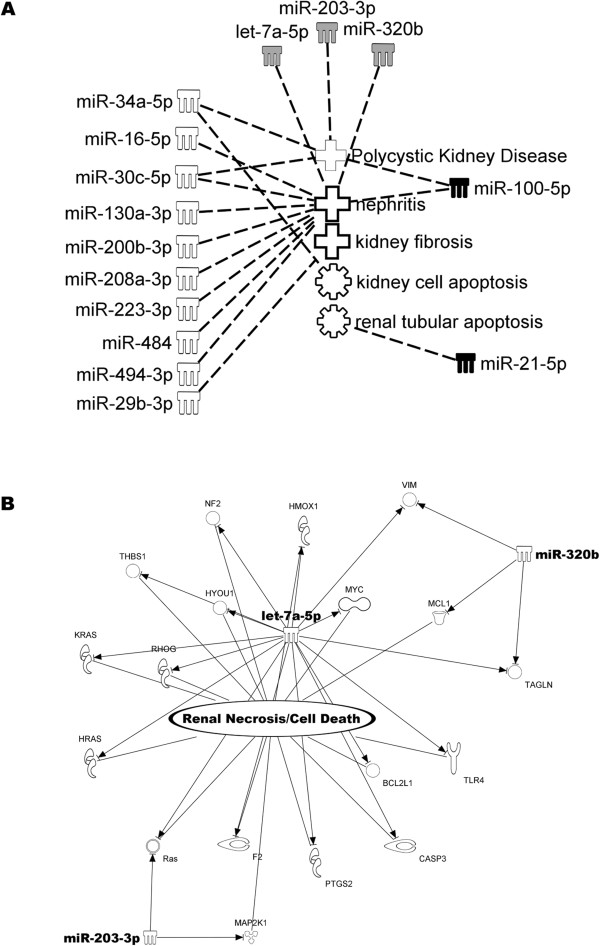
**Prediction of renal functions for miRNA from qPCR and NGS. (A)** Analysis of the urinary miRNAs with altered expression in gentamicin induced tubular injury reveals significant correlations with renal injury. Black filled symbols represent miRNAs identified by qPCR as significantly changed in renal injury. White filled symbols are miRNAs identified as significantly changed by NGS and the gray filled symbols are the miRNAs identified as changed in both approaches. Note that Ingenuity’s software combines miRNAs with a common seed sequence to a single identifier. **(B)** Potential renal disease related mRNA targets for Let-7d, miR-203, and miR-320 identified by IPA are shown.

Ingenuity modeling of the differentially expressed miRNAs identified by qRT-PCR and NGS was undertaken to better understand the targets and pathways regulated. We ran the predicted miRNA targets from the NGS and the Taqman qRT-PCR analysis separately through Ingenuity’s Tox Function prediction. The main toxicological functions associated with miRNAs detected by either platform are Renal Inflammation (pvalue < 1e-6). In addition, biological functions like cell cycle and cellular development were associated with miRNAs from both platforms. Next, pathway analysis was performed using experimentally verified targets of miRNAs from each platform. miRNAs from NGS were associated with 200 targets mainly involved with tissue injury & repair, increased levels of red blood cells, and renal necrosis. miRNAs identified by TLDA-A produced 844 targets involved in renal inflammation, kidney failure, renal necrosis, as well as increased levels of red blood cells, alkaline phosphatase and creatinine. Top pathways included IL6-Signaling (p-value < 2.9e-25) and ERK/MAPK cascade (p-value < 4.85E-11).Although multiple signaling pathways were predicted for the miRNAs from both profiling platforms, the main toxicology related function was ‘Nephritis’. Specifically, two out of the three miRNAs common to both qPCR and NGS approaches (let-7d and miR-320) mapped to this function (Figure 5A). We expanded our search for renal-related functions within the remaining set of differentially regulated miRNA’s by examining other renal-related associations within the ‘Tox Function’ and ‘Diseases and Bio Functions’ available with IPA analysis. This led to identification of several additional renal activities, including a link between the third common miRNA identified by both platforms, miR-203, and polycystic kidney disease (Figure 5A). To investigate the potential targets for the three miRNAs identified by both NGS and qRT-PCR, network analysis of the genes experimentally linked to let-7d, miR-203 and miR-320 was investigated. This analysis yielded a number of interesting genes linked to renal necrosis consistent with gentamicin induced nephrotoxicity (Figure 5B) which warrant further investigations in future studies.

## Discussion

This study builds on a growing data set indicating that miRNAs in body fluids can be used as non-invasive biomarkers for detecting and monitoring various pathophysiological conditions. Gentamicin, an aminoglycoside antibiotic used to treat many types of gram negative bacterial infections, is a known nephrotoxicant. The underlying mechanism of gentamicin renal toxicity is assumed to be mainly related to the formation of free hydroxyl radicals and the inhibition of mitochondrial respiration, although other mechanisms have also been suggested [33–36]. The microscopic findings in the kidneys observed in this study were similar to those previously documented following administration of gentamicin to rats [37–39]. Therefore, in this study, gentamicin at a dose of 50 mg/kg/day successfully induced tubular injury 7 days post treatment (Figure 1). The objective of this study was to induce nephrotoxicity in rats with gentamicin and evaluate two miRNA profiling platforms in detecting significant changes in urine samples.

Sequencing allows for complete evaluation of all small RNAs, without primer bias or a need for species annotation and as NGS analysis becomes more affordable, more scientists are choosing this platform in their investigations. Several studies have examined the concordance between various profiling platforms including quantitative RT-PCR and miRNA-seq and although it is generally believed that for high quality specimens intra-platform variability is usually minimal, cross-platform comparisons do not necessarily correlate well [21–24]. Even less is known on comparisons in biofluids, especially urine. To address this issue, we have analyzed RNA derived from urine specimen using Life technologies’ TLDA-A and Illumina’s NGS platforms. We thus prepared small RNA sequencing libraries using Illumina’s TruSeq sample preparation kit from urinary total RNA extracted and used on the qRT-PCR analysis, and sequenced the samples on the HiSeq 2000 (Figure 3). Utilizing NGS allowed us to identify several kidney-related miRNA which were significantly changed in rats exposed to the kidney toxicant gentamicin. However, the majority of miRNAs identified by NGS were not the same as those identified on TLDA-A cards (Tables 1 and 4). Thus, we report for the first time, that there is a minimal agreement between NGS and qRT-PCR for low yield urinary miRNA analysis.

The observed minimal agreement between the two platforms could be due to a variety of reasons. One reason could be the fact that we only analyzed miRNAs available on the TLDA-A, which does not include most of the miRNAs detected by NGS. TLDA allows for quantitation of 335 mouse and 226 rat miRNAs based on Sanger mirBase v. 10 annotations. Out of 375 miRNA sequences on the TLDA-A, 218 had a corresponding identifier in Rat mirBase v. 20; 147 in mouse and 1 in human. 5 TLDA-A miRNAs could not be found in mirBase v. 20 as they were deprecated. Out of the 14 miRNAs that were detected to be differentially expressed using NGS (Table 4), five (rno-let-7d-5p, rno-miR-100-5p, rno-miR-203a-3p, rno-miR-21-5p, rno-miR-320-3p) were represented on the TLDA-A, while nine (rno-miR-378a-3p, mmu-miR-5100, rno-miR-30e-3p, rno-miR-125b-2-3p, rno-miR-320-5p, rno-miR-3473, rno-miR-21-3p, rno-miR-455-5p, and hsa-miR-7641) were not. In fact, these 9 miRNAs are in their isomeric forms (Figure 4B). This finding highlights a key limitation in the qRT-PCR analysis as the TLDA primers may only detect some isomiRs with extra bases on either ends, but will miss the isomers with deletions/substitutions. Hence, only a subset of miRNAs are queried on the TLDA-A, and as our results indicate, nine miRNAs differentially regulated in NGS could not be detected by the TLDA-A due to its dependency of primer design. Employing the full TLDA array capabilities to include both A and B cards may improve concordance. The NGS platform, on the other hand, seems to be highly sensitive to the presence of contaminants. As observed in this study, contaminations ranging from PCR primers to bacterial and fungal genomes could negatively affect detectability of low expressing miRNAs. Hence the sample with the least amount of contamination contributed the maximum species of miRNAs compared to ones with higher contamination. Additionally, as depicted in Table 3, out of the total 132 and 168 rat-miRNAs detected in TLDA-A and NGS control samples respectively, miRNAs in TLDA-A seem to be far more consistently detected than in NGS.Although the absence of primer-dimer sequences indicated that there was enough genetic material to sequence, a large proportion of the reads aligned to non-coding RNAs (introns, intergenic regions, and exons). Furthermore, a large proportion (20-60%) of reads could not be aligned to the rat genome, which may indicate the presence of exogenous RNAs, mRNA degradation, genetic material expelled through urine, and other contaminants mainly from dietary, bacterial and fungal origins (Figure 4). For example, most of the miRNAs that were mapped to mouse (25) and human (2) can be traced to 3’ regions of protein coding regions of rat. Furthermore, about 0.6-16% of detected miRNAs belonged to human and mouse. 24 out of 35 mouse miRNAs and 8 out of 9 human miRNAs detected in the samples were found in unannotated regions of rat genome. These findings, together with the fact that the number of miRNAs annotated to the rat is only about a third compared to human and half as many as in mouse strongly suggests that the rat miRnome may be under-annotated.

Another reason for the low concordance between the 2 platforms may be their differences in sensitivity. Specifically, following reverse transcription of miRNA targets, we performed a pre-amplification step to increase our detection capabilities for the low expressing miRNAs, which is a step not currently available for the miRNA NGS analysis. The use of cDNA pre-amplification has been proposed to increase the sensitivity of miRNA detection, although with increased variability for low abundant miRNAs [40, 41]. However, all three miRNAs: let-7d, miR-203, and miR-320 were reliably detected by qRT-PCR in every sample with low mean C_t_ values (miR-let-7d: 27.52 ± 0.77; miR-203: 24.66 ± 0.3; miR-320: 24.69 ± 1.1) which suggests relatively high levels of urinary expression. Therefore, it is likely that although the qRT-PCR method will yield a higher level of sensitivity (especially when combined with the pre-amplification step as was performed in this study), the NGS may actually provide higher specificity. Specifically, miRNA-seq did not detect some miRNAs which qRT-PCR detected (<32 C_t_). In our study we were able to evaluate the NGS samples to a depth of 10 million reads. It would thus be interesting to examine if increasing the sequencing depth could improve detection sensitivity and concordance between the technologies.

These results suggest that although miRNA-seq is specific, due to its currently stringent RNA input requirements, it may not be able to detect certain low quantity (50 ng) and low quality (OD 260/280 < 1.8; RIN ~ 2) RNAs in urine at the conditions used in this study (50 base single end sequencing totaling 10 million reads). Additionally, although total RNA was extracted for each sample and divided between the TLDA and NGS analysis, the RNA was further enriched for small RNAs during the Illumina Truseq small RNA library preparation. Just as the pre-amplification step in the TLDA platform may introduce bias, steps such as RNA ligation may introduce technical bias during the NGS library construction [42]. None the less, as reported previously, urine in general contains very little RNA [43], and this low quality and quantity of the RNA (as shown in Table 2) might be key to the low observed concordance between these platforms. Efforts to improve the quality of RNA to be extracted from urine specimen may decrease the inter-platform variability observed. Recently, as more commercially available kits are designed for this purpose, more investigators are evaluating the quality as well as quantity of miRNAs extracted from urine [30]. These investigations together with improved methodology for enriching of small RNAs to be used in profiling platforms hold promise for improved concordance. Furthermore, although in this study we used cell-free urine probably containing both exosomal as well as non-exosomal miRNAs, enriching for exosomal RNAs may also shed light on differences in analysis between profiling platforms [30].Therefore, our data suggest that each platform has its own advantages and disadvantages and their utility should be decided based on the experimental objectives and study design limitations. Regardless of platform differences, the three miRNAs identified by both platforms, let-7d, miR-203 and miR-320, hold promise as tubular injury biomarkers due to their strong functional associations with cellular process or pathways relevant to renal disease. In fact, the main toxicology related function predicted by IPA was ‘Renal Nephritis’ (Figure 5A). Looking at each platform independently, it seems like the differentially regulated miRNAs identified by TLDA-A, namely miR-16-5p, 185-5p, 200b-3p, 208a-3p, 23a-3p, 30c-3p, 3118, 494-3p and 92a-3p which are associated with nephritis (from Ingenuity Pathway Analysis) may be promising biomarkers of tubule injury. Similarly, miR-100-5p and let-7a-5p implicated in renal inflammation and nephritis may be tubular injury biomarkers specifically detected by NGS. Taken together, these results suggest that miRNAs isolated from urine and profiled by either NGS or qRT-PCR could potentially serve as translational biomarkers for detection of some forms of renal injury.

## Conclusion

Drug induced kidney injury is not only an unfortunate clinical manifestation but it also hinders pharmaceutical progress. Urine samples have the potential to provide a wealth of information both for clinical applications as well as translational investigators, and provide a perfect medium for biomarkers of injury. To our knowledge, this is the first study comparing urinary miRNA expression data generated by qRT-PCR to those from sequencing. Although, the high cost of these experiments prohibited us from performing technical replicates and limited our sample size (7 control and 3 treated), we were able to evaluate miRNA expression analysis employing two very different technologies and show that, for at least three miRNAs, that there was high inter-platform concordance.

In summary, we have shown that differentially expressed urinary miRNAs can be detected by two distinct profiling platforms, qRT-PCR and miRNA-seq. Our data indicate that the three miRNAs detected by both technologies may be useful urinary biomarkers for tubular injury.

## Methods

### Animals

All animal experiments were conducted in compliance with the UK Animals (Scientific Procedures) Act 1986 and its associated Guidelines and Codes of Practice. Studies were reviewed internally by the Pfizer, Sandwich Ethical Review Process and subsequently authorized by the UK Home Office as Project Licence 70/6903. Male Sprague Dawley rats (225-270 g, Charles River Laboratories) were maintained in a central animal facility housed individually in polycarbonate cages with autoclaved woodchip bedding (Lillico) enriched with Lillico paperwool (nesting material). The room environment was maintained at 21°C ± 2°C and 55 ± 10% relative humidity at all times in an alternating 12-h light–dark cycle. Rats were removed from cages during surgery, toxicant injection, and blood and urine collection. Animals were acclimated to the laboratory environment for a minimum of 5 days prior to initiation of dosing. Water (purified by reverse osmosis) and Certified Rodent Diet 5002 (PMI Feeds, Inc.) were provided ad libitum. Animals were euthanized by isoflurane gas anesthesia followed by exsanguinations.

### Dosing

Gentamicin Sulphate (Sigma) [44], in a sterile solution of 0.9% saline, pH to 7 (adjusted with 1 M NaOH), was administered by subcutaneous injection once daily to male rats at doses of 0, 10, 25 and 50 mg/kg/day for 7 days (maximum of 1 mL per site and no more than 4 sites in any 24 hour period).

### Sample collection

Animals were placed into chilled metabolic cages for urine collection overnight. All animals were fasted, but provided free access to water. Total urine volume for each animal was recorded. Urine was briefly centrifuged (1000 *g* for 5 min at 4°C) to sediment any contaminants and cellular debris and was aliquoted and frozen at -80°C. Each urine protein biomarker was normalized to the total amount excreted in the volume of urine collected, and to the urine creatinine concentration (mg/ml) to normalize for dilutional effects. There was no change in the amount of creatinine excreted in the urine of control or treated animals over the time course of the studies (data not shown). KIM-1 and Beta-2-Microglobulin were measured using MSD technology, and urine protein was analyzed using Advia 1650 automated technology. Data are expressed as mean ± standard error. Statistical difference (*p* < 0.05) was calculated by one-way ANOVA. *p <* 0.05 is considered significant and is represented by a single asterisk (*) where applicable. All graphs were generated by Prism software (GraphPad Software).

### Light microscopic examination

Kidneys were weighed at scheduled necropsy, preserved in 10% neutral buffered formalin, then processed through graded alcohol and xylene, infiltrated and embedded in paraffin, sectioned, and stained with hematoxylin and eosin for histological assessment. Sections for microscopic evaluation included one coronal section through the middle third of one kidney and one mid-sagittal section through the middle third of the other kidney. Histological sections for both kidneys included cortex, medulla and pelvis. The agreed upon standardized HESI/PSTC histology lexicon were used. Histopathology evaluation was performed by a board certified veterinary pathologist who had knowledge of the treatment groups and necropsy data (organ weights and macroscopic observations) but who was blinded as to clinical pathology datasets, including results from biomarker evaluations.

### RNA extraction and quantitative Polymerase Chain Reaction (qPCR)

Total RNA was isolated from 200 μL of rat urine using a modified protocol (miRNeasy RNA extraction kit, Qiagen) according to the manufacturer’s instructions. Briefly, 700 μL of QIAzol reagent was added to 200 μL of urine. After vortexing vigorously with chloroform, the samples were then centrifuged at 12,000 g for 15 min at 4°C. The upper aqueous phase was transferred to a new tube and 1.5 volume of ethanol was added. The sample was then applied to the column and washed. The immobilized RNA was then collected from the membrane with 30 μL of RNase free water.

Total RNA concentration was measured at 260 nm using a NanoDrop 2000c spectrophotometer (Thermo Scientific). RNA concentration was further measured for urine RNA samples on a RNA 6000 Nano chip using Agilent 2100 Bioanalyzer (Agilent Technologies). This RNA was used for both the NGS as well the PCR assays. Quantitative miRNA analysis was performed using TaqMan miRNA assays from Applied Biosystems. RNA was reverse transcribed into complementary DNA (cDNA) using megaplex primers (16°C for 30 min; 42°C for 30 min; 85°C for 5 min). The cDNA product was then used in a pre-amplification step. Quantitative PCR was performed using TaqMan Low Density Array (TLDA A, Life Technologies) in a Viia7 instrument with the following temperature profile: 95°C for 10 min followed by 40 cycles of 95°C for 15 s and 60°C for 1 min. Only miRNAs that were detected with a C_t_ value of 32 or less and in at least 20% of all the samples were included in the analysis. We investigated several normalization methods, including invariant miRNAs, global mean, and lowess using the Expressionist version 7.6 (Genedata). Welch test was applied to each normalized data set to assess differential regulation between control and treated samples. All three normalization methods yielded similar results in terms of statistical significance (P-value < 0.05) and fold changes (FC magnitude > 1.5). However, we report the results derived from the lowess normalization as it produced the best match to the NGS data. All graphs were made by Spotfire DecisionSite (Spotfire Inc.). The miRNAs from TLDA-A, which are based on mirBase v.10, were mapped to mirBase v. 20 using their corresponding v.10 sequences. This allowed us to focus our efforts on the v. 20 miRNA sequences in our comparative analysis between TLDA-A and NGS.

### NGS analysis

miRNA reads were obtained from BGI. The 3’ and 5’ adaptors were trimmed off the reads and sequences between 16 and 35 nucleotides of average phred score of ≥ 30 were retained for further analysis. The reads were first mapped to rat miRNAs using bowtie [45] without allowing for mismatches. Instead of discarding the remaining reads, we explored the possibilities of RNA editing to include potential isomiRs [46]. The reads were thus mapped to miRNAs that have at most 1 base mismatch, a substitution at the 5’ or 3’ ends, or have 1 or 2 extra bases at either ends. Although 1 base mismatch would include 1 base substitution/deletion/addition at the ends, we made sure that 1 base mismatch was inside the miRNA and that all categories were mutually exclusive. To account for heterogeneity, reads were further mapped to the precursor miRNAs. Reads that uniquely mapped to miRNAs were considered and were profiled as: 1) identical to miRNAs, 2) perfectly match to miRNAs but are of shorter length (no more than 5 bases short), 3) mapping to miRNAs with 1 substitution, 1 or 2 extra bases at 3’ and 5’ ends and with those with at most 1 mismatch (occurring inside the sequence and not on ends), 4) mapping to the precursor miRNA. Thus, steps 2–4 constitute “IsomiRs”-alternative forms of the mature miRNAs. We merged the counts of reads identical to a miR and those mapping to Isomeric forms. Additionally, since the total number of annotated mature miRNAs in rat is 728 compared to 1908 in mouse and 2578 in human, we accounted for the possibility of miRNA under-annotation in rat by expanding our mapping to include miRNAs from mouse and human. Thus, after aligning to rat, the remaining reads were further mapped to mouse and eventually to human miRNAs. MirBase v. 20 was used for miRNA sequence mapping. Since urine samples may contain exogenous RNAs and other small RNAs, reads were also mapped to the RFAM (RNA Families) database [47] and exon sequences in the rat. The other common contaminant in miRNA-Seq is primer-dimers which can adversely affect the quantification of miRNA abundance. Specifically, the Illumina protocol consists of manual gel band excision, which enhances the occurrence of primer-dimers due to incorrect cutting of the gel or insufficient sample to sequence. We thus included extensive primer-dimer analysis in our study. Furthermore, since normalization and differential expression analysis in mRNA-Seq are still being finalized by the scientific community, we employed three different widely used methods in our analysis. We first obtained a Reads per Million (RPM) count for a miRNA by dividing the number of reads mapping to that miRNA by the total number of reads mapping to all miRNAs and multiplying that by 1,000,000. This gave the abundance of a miRNA in a million reads. Additionally, the counts were normalized using scaling factors assuming a negative binomial distribution using DESeq (Differential Expression for Sequence Count Data) [48] and trimmed mean normalization using EdgeR (Empirical analysis of Digital Gene Expression in R) [49] packages in R-Bioconductor. We next used various filtering criteria and hierarchical clustering to achieve a clear segregation between the control and treated samples. Spearman correlation coefficient was used on the normalized values and a “complete” hierarchical clustering was then performed. To find the optimum differential analysis we investigated three different methods [50]: Limma, variants of Fisher’s exact test from DESeq, and EdgeR packages from the Bioconductor software. A p-value cutoff of 0.05 was used for significance detection. In addition, to check for sequences that are of dietary origin, all unaligned sequences were aligned against wheat (*Triticum aestivum*), corn (*Zea mays*) and soybean (*Glycine max*). Sequences were first mapped to wheat, then the unaligned sequences were mapped to corn and then to soybean. The miRNAs from human and mouse that were detected in the rat samples were blasted against the rat database in NCBI. Since it also consisted of miRNAs in only isomeric forms, blast results that had >85% of the sequence aligned were considered for further analysis.

### Prediction of toxicity effects for NGS/TaqMan significant miRNAs

The 14 miRNAs identified as significant by NGS and the 32 miRNAs identified by TaqMan were analyzed separately in Ingenuity System’s Ingenuity Pathway Analysis IPA tool. IPA Core Analysis was performed on each set and the most significant ‘Tox Functions’ along with relevant biological functions are reported. Experimentally verified gene targets were then further evaluated using IPA’s ‘Core Analysis to extract over-represented biological pathways with p-value < 0.01. Network analysis was performed on the three conserved miRNAs (let7a-5p, miR-203-3p, and miR-320b) using IPA’s ‘Grow’ function to add genes experimentally linked downstream of the miRNAs via ‘RNA/RNA interactions analysis: miRNA targeting’ relationship. The resulting network was expanded using the ‘Overlay’ function with IPA’s ‘Ingenuity Tox List’. The most overlapping list ‘Renal Necrosis/Cell Death’ was added and the unlinked genes trimmed off for better visualization.
